# A Contribution to the Study of Malignant Diseases

**Published:** 1907-09-21

**Authors:** Skene Keith, Geo. E. Keith


### Hospital Clinics.

### A CONTRIBUTION TO THE STUDY OF MALIGNANT DISEASES.

By SKENE KEITH, F.R.C.S.Ed., and GEO. E. KEITH, M.B., C.M.

The treatment of malignant disease may be divided into two methods—the empirical and the scientific. We propose here to deal entirely with the former, as we have been experimenting more or less continuously since 1886 in this direction, and we seem at last to have made some slight but definite advance.

The empirical method of treatment consists either in simply experimenting with anything—radium, for instance—or in thinking out some working hypothesis. For some years we have worked on the idea that as there is always present in malignant disease a marked deterioration of the blood, good results might be obtained if we could improve the condition of the blood up to, or even beyond, the normal. Of the many difficulties met with there are two which perhaps hamper one most. First, the difficulty that we have very little knowledge to start with. We do not know why the disease attacks one person and not another; we are uncertain whether the disease is hereditary, and whether it is local or general. There seems, in fact, scarcely one point in common about different cases, except that if the disease be not arrested, the patient must surely die. The second difficulty is that empirical treatment can only be carried out either on patients who come to us after surgery has failed, or where the disease is situated in a non-surgical part of the body.

One of the drugs with the oldest reputation in the treatment of cancer is arsenic. When the hypodermic method of administration came into use, we found that arsenic given in this way seemed to have a more beneficial effect than when given by the mouth. Nearly four years ago we tried a mixture of arseniate of iron and Merck's iodipin. With this mixture we found that pain was in the great majority of cases relieved after a few injections. We began by using ordinary doses, but got on better when we doubled the amount of arsenic. We doubled again, and then again, but as the last increase in strength did not seem to be an improvement, we went back to the third strength. We have been much indebted to Mr. Charles N. Caines, of Messrs. Squire and Sons, for making our various solutions. In the use of cinnimate of sodium, which was recommended by Dr. Drage some years ago, we have been disappointed. This drug is, like arsenic, one of the old cancer remedies, and, although we did not obtain any good results from its use alone, we found that cases did better when it was added to the arsenic and iodipin mixture. To this mixture, which is a mechanical one and makes a thick yellow emulsion, we have added a large number of other drugs; but we have come back to the emulsion of arseniate of iron, cacodylate of iron, cinnimate of sodium, and iodipin as being the most effective. The exact mixture consists of iodipin, 25 per cent., ʒj.; arseniate of iron, ♏xx.; cacodylate of iron, ♏xx.; cinnimate of sodium,, ♏xx. Of this we inject up to 5 c.c., sometimes twice a week, sometimes every day. The amount given per week is still a matter for experiment; at present our maximum is 30 c.c. in the course of a week. The cacodylate of iron contains 3 grs. of iron in 1 c.c.; the arseniate of iron ¼ gr. of iron and $\frac{1}{45}$ gr. of arsenious anhydride in 1 c.c.; the cinnimate of sodium is a saturated solution, containing 1½ gr.

We have treated in all between sixty and seventy cases, and in the great majority of these there has been more or less relief of symptoms and increase in the probable expectation of life. The symptom common to so many cases of cancer—pain—has been relieved in a way which seems impossible to explain. In one case where the patient, a lady of 64 years of age, had suffered torture—and she was emphatic that this word alone described what she had endured—the pain was relieved almost entirely after the first injection.

In carrying out this treatment the age of the patient and the state of the pulse are the most important considerations. If the patient is over 60 years of age, or if the pulse runs at much over a hundred a minute, it is very unlikely that treatment will do good. Again, if the pulse is about a hundred, and does not come down to eighty after a month's treatment, or if the patient looks much older than his years, the prospect is very decidedly unfavourable.

We give below an abstract of eleven cases, suppressing, for the sake of space, all details as to family history, etc., not bearing on treatment.

Case I.—Mrs. L., æt. 60, large ulcerated scirrhus of breast, was seen on May 19, 1903. Duration of disease, six months. Had attended the Cancer Hospital for one month, and was told that no operation was advisable. At Charing Cross Hospital, where she had gone the week before, her friend was told that the case was hopeless, and that the patient could not live for more than six weeks. Severe pain and increasing debility had caused her to give up work 4½ months ago. She had had rheumatic fever twice, had a loud aortic regurgitant murmur, and suffered much from chronic bronchitis. The growth measured 8½ in. by 7 in., and projected so much into the axilla that the arm could not be brought to the side. There was an ulcerated surface 2 in. by 1½ in. After the first injection we were much surprised to be told that there had been great diminution of the pain. This was an entirely voluntary statement, as we did not expect any such result. In one week, the injections being given every day, the pain had entirely disappeared. On July 4 the patient was able to go back to her work as domestic servant, but on account of shortness of breath she had to leave on August 27. On the 29th there were symptoms of heart failure, and death occurred two days later. The doctor who saw her considered death was due to the condition of the heart. Sixty-five injections were given in all, and the mass of disease had diminished by about one-half.

Case II.—Æt. 35, was sent by Dr. Russell Wells on September 24, 1903, with an ulcerated scirrhus cancer of the breast. Duration of the disease, one year. The glands in the axilla were enlarged. The patient was getting rapidly thin. Fifty-nine injections were given by May 3, 1904, and by August not a trace of disease could be felt. There has been no return, and the patient is in the best of health (June 1907).

Case III.—Æt. 65. Seen first in June 1904. The right breast had been removed in May 1903 and a fourth operation had been performed in May 1904—four operations in thirteen months. There was some fullness in the axilla above and below the clavicle. The symptoms were pain and swelling of the arm. Thirty injections relieved the symptoms, but in the following January a small nodule appeared below the clavicle. The injections were repeated. In May the lower ribs were much pushed out, and the note on percussion was quite dull. Eighteen injections cleared up the dullness and got rid of the bulging of the chest. In August a hard growth in the skin, the size of a shilling, situated low down on the chest wall, was removed. In November there was one nodule above and two below the clavicle, and the patient was persuaded to have *x*-rays in addition to the injections. June 1907, a course of injections and *x*-rays has been repeated four times in the past eighteen months, and the disease is almost kept in check—almost, but not quite. There is now very little swelling of the arm.

Case IV.—Æt. 59, was seen in July 1904. The right breast had been removed four years before, and the disease had returned in the scar after an interval of three years. High frequency had been tried to relieve pain, without result. The patient was much emaciated and complained of great pain. The pulse was feeble at 112. There were several nodules adherent to the scar and to the ribs, with enlarged glands in the axilla and neck; there was also marked bulging of the ribs and complete loss of resonance. The patient was so feeble that she was advised to go home, but she begged for the chance of relief to the pain, if nothing else could be hoped for. Partial relief came after the first injection, and freedom from pain after the tenth. In every way there was steady, if slow, improvement, and the patient went home to Scotland after forty-three injections. She had put on a little weight (8 lb.), felt fairly well and much stronger; the dullness of the chest had entirely disappeared, and there was only one nodule to be felt, the remainder and the enlarged glands having entirely disappeared. The pulse kept from 72 to 80. The patient was to return for further treatment in two months' time, but wrote to say that as she was feeling so well she did not think it necessary. She kept well till March, when she got an attack of bronchitis, to which she was subject, and died suddenly, apparently of heart failure.

Case V.—Æt. 63, was first seen in July 1904. In the preceding October a lump was first noticed in the breast, the arm swelled in January, and in March the red mass to be described appeared. On examination a large, hard mass taking in practically the whole breast and running continuously to the top of the axilla was found. On the breast there was a projection of a dark purple colour, about two inches in diameter. The hand and arm were much swollen and painful. Operative treatment, though possible, was not favourable; it was positively declined, and injections were agreed to instead. Contrary to our usual experience, the injections gave a great deal of trouble, hardly one being given without causing pain and swelling. The patient had lived much in the East, and stings from insects had always given her more trouble than most people. By the end of September twenty-seven injections had been given, with the result that the growth was very much smaller. In November the patient was alarmed by a severe hæmorrhage from the purple-coloured swelling, and, as the skin had become destroyed over it, she consented to have the breast removed. This was a very much less formidable operation than it would have been in July, and it was specially noticeable for the very small amount of bleeding. Subsequently the patient became jaundiced and died in March.

Case VI.—Æt. 50, an alcoholic, had a large scirrhus cancer of the breast removed in hospital in December 1902. There was recurrence in eighteen months, and the following is the surgeon's report: "Extensive recurrence, not only beneath the scar and adherent to bone, but nodules inside the scar and also in the skin of the axilla. I cannot advise further operation with any hope of success." Twenty-two injections were given between October 17 and January 7, 1905. The pain was completely removed, and very little growth could then be felt. General peripheral neuritis was diagnosed in March, the pain being then all over the body, and the patient died on April 12. At the post-mortem examination the body was found to be well nourished. All the growth, except one small nodule in the skin of the axilla, was removed. It was the size of a small hazel nut, was not adherent to the bone, and presented the characteristics of ordinary scirrhus cancer.

Case VII.—Æt. 75, was sent by Dr. Gardiner, of Richmond, on July 27, 1903. Three inches inside the anus the rectum was almost closed by a large cauliflower growth springing from the whole circumference of the bowel. Nothing could be passed except fluid motions. In ten weeks the patient put on 23 lb. in weight, and three fingers could easily have been passed through the growth. In January about half the circumference of the bowel was free, and large, solid motions could be passed without difficulty. On February 22, after walking four miles, he hurt his back lifting a heavy tub, and was confined to his bed for six weeks. He never thoroughly recovered from this accident, constantly complaining of pain in his back. Towards the end of September two patches of senile gangrene appeared on the heels, and, spreading, caused death on October 22.

Case VIII.—Æt. 59, was seen on June 27, 1904, suffering from stricture of the œsophagus low down. Difficulty in swallowing was first noticed in April. Now very small quantities of fluid can, at times only, be got into the stomach. Six months ago he weighed 14 st. 3 lb., and has now been reduced to 8 st. He had been a heavy drinker all his life. The pulse kept about 96. It was explained that treatment was practically a race to see if the obstruction could be relieved in time to stop the starvation. Ten injections were given, with the result that the patient could drink milk freely. Unfortunately, having little or no self-control, he would drink till he was sick, bringing up large masses of curd. This naturally increased the exhaustion, and he died on July 19.

Case IX.—Æt. 63, was seen with Dr. Pearson in July 1903. She had noticed a swelling in the left hypochondriac region about a year before, and a short time afterwards had to give up her calling of a sick nurse on account of increasing debility. She was also very anæmic, wasted, and was much constipated. A large tumour distended the abdomen, filling the whole of the left side and extending fully four inches across the middle line. Some five or six doctors had seen the case, but no agreement as to the diagnosis had been come to, except that the tumour was malignant. Seventeen injections were given. The tumour rapidly decreased in size, but the patient did not feel better, and treatment had to be discontinued. The old lady died in September, and Dr. Pearson wrote: "The growth had very markedly diminished in size, and though she was very much more emaciated, no sign of it could be seen externally, the abdomen being perfectly flat. The growth proved to have primarily arisen, I should surmise, in the descending colon, which was adherent to the parietal peritoneum. It had undergone considerable degeneration, and was very friable. I cannot express to you how surprised I was to find the bulk of it so much diminished. The lumen of the gut was perfectly patent." The microscope report said: "The larger section (colon) is infiltrated with a degenerated colloid carcinoma, which is probably of the columnar-celled type, though its cells are now much altered."

Case X.—Æt. 50, was seen by Miss Gertrude Keith on January 13, 1904, and treated by her. Cancer of the liver had been diagnosed by several doctors. Jaundice was noticed in the previous May. The skin and conjunctivæ were very deeply jaundiced, the weight was 7 st., 8 lb., the stools were clay-coloured, and the urine was almost black. She complained of increasing debility, loss of weight, great pain in the liver, and very intense irritation of the skin. She was too weak to go out alone. The liver was enlarged and hard, extending from the fourth rib to below the umbilicus, with an irregular and hard projection in the position of the gall-bladder. The patient had always been most temperate. Treatment was recommended more with the hope of gaining some information than with the expectation of doing much good. By the 21st of the month the pain was relieved. Five days later there was a sharp attack of diarrhœa, caused probably by the nuclein which we were using at the time. This was followed by some relief to the itching. On February 27 the liver extended from the sixth rib to two inches below the ribs, the mass in the position of the gall-bladder being apparently larger. On March 5 a slight tinge of yellow was noticed in the motions, for the first time since September. The case is much too long to report fully. The eighty-second injection was given on May 20, 1904, the patient then being well and free from jaundice, with stools and urine normal. Sixteen additional injections were given before the end of the year. Only seventeen were given during the whole of the following year. The weight had gone up to 8 st. 7 lb.—a gain of 13 lb.

In the beginning of 1906 there was a return of all the old symptoms, accompanied by rapid emaciation; weight, 7 st. 1 lb. in April, a loss of 20 lb. Twenty-three injections were given, but by July she was thinner and weaker. She had fourteen injections in September and October, and by December was putting on flesh, was hardly jaundiced, and was able to do her housework and washing. The weight in December was 7 st. 7 lb. In June 1907 she was, apparently, quite well. This case is a good example of the absolute necessity of never giving up hope, but of persevering obstinately in the face of every discouragement.

Case XI.—Æt. 63. In the summer of 1904 a hardness was noticed round a scar which had been made six years before, when gall-stones were removed. This hardness was cut away on August 5, and pronounced to be a sarcoma of most virulent type. In September glands were removed from both groins and from the right axilla. A third operation was performed for recurrence in the left groin on November 9, but though the operation was an extensive one, the whole of the growth could not be removed. The acuteness of the disease was shown by the fact that three operations had to be performed within one hundred days, and even in that short time the disease had got beyond the reach of surgery. Before the wound had healed nodules of the disease had spread almost up to the umbilicus and on to the thigh and left labium. The patient was confined to bed, and the general condition was so bad that the surgeon who had operated gave her three months to live at the outside, and greatly feared that the death would be a most painful one. The patient was first seen on November 20, and by February 9 thirty injections had been given. Improvement did not begin until after the twelfth. After the thirtieth she had gained nearly two stone in weight, and was able to live her ordinary life. In the beginning of April the right arm swelled, and some thickening was found in the axilla. The injections were recommenced, and a second thirty had been given by August 7, when no trace of disease could be found. The patient went abroad until the beginning of October, coming back apparently quite well. At the end of the month she complained of pain in the right groin, the leg and thigh became swollen, and a mass could be felt on deep palpation of the abdomen. The injections now did not have any effect, and the patient died on December 13 partly of the disease and partly from bronchitis, although without the bronchitis she would not have lived very long.

Results such as we have given may not seem to be extraordinary, but to be able to do anything at all in cases of cancer is worth something, and we trust that these observations will induce others to try something on the same lines with the object of relieving the pain of malignant disease. Surgery calls out loudly and rightly for early operation, but we feel that the majority of surgeons are too apt to pin their faith to surgery alone, and are not sufficiently energetic in advising, or at least allowing, a trial of other auxiliary methods of treatment such as that described above.

